# Molecular Identification of New Cases of Human Dirofilariosis (*Dirofilaria repens*) in Italy

**DOI:** 10.3390/pathogens10020251

**Published:** 2021-02-23

**Authors:** Simona Gabrielli, Valentina Mangano, Federica Furzi, Alessandra Oliva, Serena Vita, Roberto Poscia, Paolo Fazii, Josephine Di Paolo, Raffaella Marocco, Claudio Maria Mastroianni, Fabrizio Bruschi, Simonetta Mattiucci

**Affiliations:** 1 Department of Public Health and Infectious Diseases, Sapienza University of Rome, Piazzale Aldo Moro 5, 00185 Roma, Italy; f.chicca95@gmail.com (F.F.); alessandra.oliva@uniroma1.it (A.O.); claudio.mastroianni@uniroma1.it (C.M.M.); simonetta.mattiucci@uniroma1.it (S.M.); 2 UOS Parasitology, AOU Policlinico Umberto I, Viale del Policlinico 155, 00161 Rome, Italy; 3 Department of Translational Research, School of Medicine, University of Pisa, 56126 Pisa, Italy; valentina.mangano@unipi.it; 4 Unit of Microbiology, Pisa University Hospital, 56126 Pisa, Italy; 5 INMI IRCCS Lazzaro Spallanzani, 00149 Rome, Italy; serena.vita@gmail.com; 6 Clinical Research Department, Direzione Generale AOU Policlinico Umberto I, Viale del Policlinico 155, 00161 Rome, Italy; R.Poscia@policlinicoumberto1.it; 7 S. Spirito Hospital, Via Fonte Romana, 8, 65124 Pescara, Italy; paolo.fazii@tin.it (P.F.); dipaolojosephine@gmail.com (J.D.P.); 8 S. Maria Goretti University Hospital, Via Antonio Canova, 04100 Latina, Italy; raffa.mar81@gmail.com; 9 Programma Monitoraggio Parassitosi, AOU Pisana, Via Paradisa 2, 56126 Pisa, Italy

**Keywords:** *Dirofilaria repens*, humans, Italy, molecular diagnosis, mtDNA *cox*1

## Abstract

(1) Dirofilariosis is a vector-borne parasitic disease mainly in domestic and wild carnivores caused by *Dirofilaria* (*Noctiella*) *repens*, which is endemic in many countries of the Old World, and *D. immitis*, which has a worldwide distribution. In recent years, an increase in the number of human cases has been reported, suggesting that dirofilariosis is an emergent zoonosis. Here, we describe further cases (N = 8), observed in Central Italy during the years 2018–2019. (2) Molecular diagnosis was performed on: (i) live worms extracted from ocular conjunctiva, cheek, and calf muscle; (ii) histological sections of surgically removed nodules from parenchymal lung, coccyx, and breast. (3) Sequence analysis (650-bp) of the mitochondrial cytochrome oxidase subunit I gene (mtDNA *cox*1) showed a match of 100% with the sequences of *D. repens* previously deposited in GenBank. ELISA test to detect IgG against filarial antigens was performed on four patients’ sera and resulted positive in two patients who showed ocular and subcutaneous dirofilariosis, respectively. Microfilariae have been never detected in the peripheral blood of the patients. (4) The occurrence of N = 8 new cases of human *D. repens*-infections observed in a two-year period suggests an increased circulation of the parasite in Italy. Therefore, dirofilariosis should be included in differential diagnosis in patients presenting subcutaneous and/or pulmonary nodules. Molecular diagnosis of the etiological agents is fundamental. Specific serological diagnosis needs to be improved in future research work.

## 1. Introduction

Dirofilariosis is a vector-borne parasitosis caused by filarial nematodes (i.e., species of the genus *Dirofilaria*) transmitted to domestic and wild carnivores by mosquitoes belonging to the family Culicidae. After mosquito bloodsucking, the infective larvae L3 penetrate into the skin and, in animals, become adult worms producing circulating microfilariae in the peripheral blood. Humans are accidental dead-end hosts of *Dirofilaria* spp. as in most of the reported cases, the infective larvae perish before attaining worm maturity. In Europe, where the two species of *Dirofilaria* spp. (i.e., *D. immitis* and *D. repens*) are co-endemic, it seems that the majority of human cases have been attributed to *D. repens* [1,2]. However, few confirmed cases of *D. immitis* have also been reported [3,4]. Conversely, the latter species is well known to be the causative agent of many cases of human dirofilariosis in North America [5] and in Japan, where *D. repens* is not endemic [6,7]. The different frequency of human infection due to *D.immitis* in different geographical areas suggests that this species would be a complex of cryptic species or that, at least, population-specific differences would exist having also differential zoonotic potential [5]. Cryptic species or intraspecific genetic variation might also exist within the taxon *D. repens*. In this regard, a fixed nucleotide variation detected in the sequences of the 18S-ITS1-5.8S region allowed to suggest the existence of a novel *Dirofilaria* species, i.e., *D. hongkongensis,* responsible for both human and canine infection in Hong Kong [7,8]. In addition, the comparison of complete mitochondrial genome obtained from *D. repens* from Europe and *Dirofilaria* sp. “*hongkongensis*” from India suggested the existence of another possible species/genotype, provisionally indicated as *Dirofilaria* sp. “Thailand II” [9].

The bloodsucking arthropods *Aedes albopictus* and *Culex pipiens* are the most important vectors of *D. repens* and *D. immitis* in several European countries, including Italy. They are efficient vectors transmitting the infection also to humans [10]. Humans develop abortive infections or syndromes due to migrating larvae that cause pulmonary nodules, ocular pathologies, or subcutaneous lesions [11,12], which could be initially misidentified as malignant tumours, requiring invasive procedures and surgery to achieve correct identification. Diagnosis is usually based on the morphology of removed worms, followed by molecular confirmation through amplicon sequencing of target loci [13,14,15,16].

In recent years, clinical cases of dirofilariosis have been increasingly reported in both animal and human hosts due to several triggers, such as the improvement in the network of pathological services and the refining of the diagnostic tools [17]. Additionally, it is well-known that change in climatic conditions (temperature, relative humidity, rainfall, evaporation) would favour both the development of the mosquitoes and of the larval phase of filarial parasites inside the vectors [18]. Indeed, more than 3,500 human cases due to *D. repens* and 25 to *D. immitis* have been reported in Europe from 1977 to 2016 [19].

Italy is traditionally endemic for canine and human dirofilariosis and it is one of the countries with the greatest number of human cases so far described [18,20]. Indeed, the number of human dirofilariosis due to *D. repens* reported in the literature after the first observation by Addario in 1885, amounted to about 410 in 1995, 181 of which had occurred in Italy. In 2001, Pampiglione and collaborators described further 60 cases occurring between 1990 and 1999 [11].

Considering that only superficial infections can be easily detected, the actual number of infected subjects is likely to be higher than what is reported, suggesting that dirofilariosis is an underestimated, emergent zoonosis.

Supporting this hypothesis, here we describe eight new cases of human dirofilariosis that have come to our attention in the last two years in different Hospitals of Central Italy.

## 2. Results

### 2.1. Patients and Materials

The molecular analysis for the identification of the zoonotic species was carried out on: (i) live worms obtained from 2 cases from ocular conjunctiva, 1 from calf muscle and 1 case from cheek; (ii) on histological sections of the surgically removed nodules from lungs (2 cases), coccyx and breast (Table 1).

### 2.2. Clinical Manifestations

**Figure 1:**
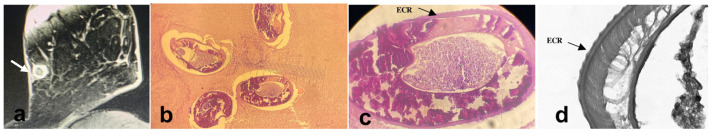
(**a**) A computed tomography scan showing a nodule in the left breast (case 8); (**b**) Cross-sections of *D. repens*, showing different diameters in the same subject, cut at different level of the worm; external cuticular ridges (ECR) are well visible (40×, H/E) (case 2); (**c**) A closer view of the section and of the typical longitudinal ridges of *the D. repens* cuticula (**d**) (case 2).

Patients reported different clinical manifestations depending on the localization of the worm. Patients with pulmonary nodules (n = 2) were asymptomatic and the lesions were initially misidentified as malignant neoplasm, thus requiring invasive investigations before the correct diagnosis. Patients with ocular nodules (n = 2) reported redness, swelling, and intense itch on both eyelids of the right eye and the nematode which appeared fleetingly and visible under the conjunctiva. Patients with subcutaneous nodules (n = 4) experienced local erythema, pruritic dermatitis, urticarial manifestations, and swelling with a rapid and painless extension of the mass; nodules were located at varying depths and showed elastic consistency to the touch and slightly painful on palpation (Figure 1a).

### 2.3. Laboratory Diagnosis

The primary examination of the surgical specimens revealed nodular lesions of soft consistency with areas of necrosis, and histological exams confirmed extensive necrosis surrounded by chronic inflammatory reaction. The main finding consisted of the presence of worms embedded in the necrotic material, showing a thick cuticle and internal organs and exhibiting morphological features ascribed to filarioid nematodes.

The nematodes that were entirely removed from the eye, cheek, and breast were sexually immature females, cylindrical, whitish, from 11 to 14 cm in length and 0.3 to 0.4 cm in width (Figure 1b). Microscopy revealed a thick laminated cuticle with the characteristic longitudinal ridges and cross-striations. The anterior end was bluntly rounded and was of greater diameter than the posterior end. The body cavity contained a female reproductive system, with the bulbous vulva of about 1 mm from the anterior end and ended in a uterine bifurcation (Figure 1b). Thus, macroscopic and microscopic features allowed a preliminary identification of the worms as *D. repens*-like specimens (Figure 1c,d) [21]. Morphological identification was then confirmed by the sequence analysis of the mtDNA *cox*1 obtained from the worms, showing 100% similarity with respect to the sequences at the same gene locus of *D. repens* previously deposited in GenBank, under the accession numbers MT847642.1; MT683122.1; KF692102.1; MF695085.1. The last sequences were obtained from specimens isolated in humans, dogs, and mosquitoes of different European countries. The sequences obtained from this study were deposited in GenBank under the following accession numbers: MW525256, MW525257, MW525258, MW525259, MW525260.

The ELISA test was performed on 4 patients’ sera and it resulted positive in 2 cases with ocular and subcutaneous dirofilariosis. Finally, microfilariae were not detected in the peripheral blood of the patients.

## 3. Discussion

Despite humans being accidental hosts for both *Dirofilaria* species, most of the human cases described worldwide have been attributed to *D. repens*, which shows a higher zoonotic potential in comparison to *D. immitis* [19].

Here, we describe eight further cases of human dirofilariosis caused by *D. repens* observed over two years in Central Italy. The most frequent site of the lesions, in line with that reported in the world literature and in previous Italian case studies, is in the subcutaneous tissue [1,11], which occurs also in disparate locations, such as the calf muscle and the cheek.

The frequent diagnosis of this zoonosis during a such relative brief period (two years) from a restricted group of researchers working in Central Italy provides evidence that dirofilariosis is an underestimated, but emerging, zoonosis in the study area. This evidence is also supported by the observed increasing circulation of the infection in canine population throughout Europe, including Italy [2]. Several factors have been well-documented to favour the transmission of both *Dirofilaria* species throughout the old world. Furthermore, *D. repens* is showing a faster and more intense dissemination through Europe [18,19,22,23] since the absence of clinical signs in most canine infections and limited options for adulticide treatment make it more difficult to effectively control this species compared to *D. immitis* [2].

Nonetheless, despite Italy being the country with the highest number of human clinical cases in Europe [11,20,23], a general lack of awareness of this parasitic disease persists.

In the diagnosis of human dirofilariosis, indirect methods are not useful as microfilariae—the stage mainly responsible for triggering an immunological reaction in infections with filarial nematodes in humans—rarely occurs in human dirofilariosis and, accordingly, antibodies against filariae cannot be detected in the majority of patients [24]. In addition, specific serological tests for dirofilariosis have not been yet developed [18]. In this study, microfilaremia was not detected in any of the reported cases, not even in those patients who resulted positive to IgG immune response.

In addition, the lack of circulating microfilariae does not allow the use of parasites DNA detection in patient’s peripheral blood samples as a possible tool for diagnosis. Therefore, most reports of human dirofilariosis have been mainly based on histopathological findings of extracted nodules. Examination of microscopic morphological features of human dirofilariosis should indeed be considered when examining solitary nodules of uncertain nature in subcutaneous tissues or mucous membranes. This is of prime clinical importance when patients reside in areas where high infection rate in dogs is reported.

Methods of molecular diagnosis, based on direct sequencing of the parasites’ DNA extracted from nodules or on entire/fragment of the worm, as in the cases here described, are of fundamental importance to identify the etiological agent involved. In the present study, the mtDNA *cox*1 locus was chosen for the genetic characterization of the *Dirofilaria* isolates, because the sequence analysis at this gene locus can reliably differentiate between different species of filariae and it had been previously used for *Dirofilaria* species identification [25]. Sequences of the mtDNA *cox*1 in the present human isolates of *D. repens* have not shown nucleotide differences among themselves, nor with respect to the homologous sequence of the parasite species previously deposited in GenBank. Future genetic investigation on populations of *D. repens* and *D. immitis*, from both natural and accidental hosts (humans) carried out by a multilocus genetic approach, involving both mitochondrial and new nuclear markers, is envisaged in order to detect the possible existence of genetic heterogeneity within those species and to define their population genetic structure, as previously suggested [9]. In addition, such analyses would be helpful to identify the geographical origin of isolates in new endemic areas and, therefore, to track spreading of the infection. In addition, a genome-wide analysis [26] of those species can identify the existence of cryptic species or subpopulations that would also differ in their biological properties, including important epidemiological features, such as a differential zoonotic potential, preference for a certain vector species, host resistance, and likely a differential specific antigenic pattern.

## 4. Conclusions

The occurrence of N = 8 new cases of human dirofilariosis observed in a two-year period suggests an increased circulation of the *D. repens* in Italy. Therefore, this parasitosis should be included in differential diagnosis in patients presenting subcutaneous and/or pulmonary nodules and a molecular test should be performed to achieve a more accurate etiological diagnosis. Specific serological tests need to be improved in future research work, as well as molecular markers to detect possible existence of cryptic species or subpopulations within *D. repens*.

## 5. Materials and Methods

### 5.1. Patients and Materials

N = 8 human patients suspected of dirofilariasis infection involved in the present study were observed between 2018 and 2020 directly by parasitologists and/or specialists in infectious diseases, working in various hospitals in central Italy.

Single subcutaneous nodules or migrant worms were removed from the cheek (case 1), the parenchymal lung (case 2, 3), calf muscle (case 4), ocular conjunctiva (cases 5, 6), coccyx (case 7), and breast (case 8) in patients referred at the Policlinico Umberto I Academic Hospital, Rome (cases 1–5), Pisa Hospital (case 6), Pescara Hospital (case 7), and Latina Hospital (case 8).

### 5.2. Histological Analysis

The excised nodules were fixed in buffered formalin (10%) and routinely processed. The histological sections, stained with haematoxylin–eosin, were first microscopically examined (Nikon SE, 40× magnification) to recognise a nematode assigned to the genus *Dirofilaria* according to morphological features, as previously described [11,27].

### 5.3. Molecular Analysis

The molecular diagnosis was performed on DNA extracted from histological sections or 2 mg of the entire worm for each patient, using the QIAamp DNA Kit (Qiagen, Hilden, Germany) following the manufacturer’s instructions.

A fragment of about 650-bp of the mtDNA *cox*1 locus was amplified by Polymerase Chain Reaction (PCR) using the primers COIintF (5’-TGATTGGTGGTTTTGGTAA-3’) and COIintR (5’-ATAAGTACGAGTATCAATATC-3’) [28,29]. PCR was performed in a final volume of 25 μL under the following final conditions: 10X buffer including 1.5 mM MgCl_2_, 0.2 mM of each deoxynucleotide triphosphate (dNTP), 1 mM each of forward and reverse primers, and 1 unit of polymerase (BIOTAQ, Bioline, UK). To test the specificity of the reaction, 2 μL of DNA extracted from *D. repens* and *D. immitis* and equivalent volume of double distilled water were included in each PCR run as positive and negative controls, respectively. The amplification was performed in a thermocycler (BIO-RAD, Des Plaines, IL, USA) using the following cyclic profile: initial denaturation at 94 °C for 10′, followed by 5 cycles of further denaturation at 94 °C for 30″, annealing at 52°C for 45″ and an extension at 72 °C for 1′, followed by 30 cycles of further denaturation at 94 °C for 30″, annealing at 54 °C for 45″ and an extension at 72 °C for 1, followed by a final extension for 7 min at 72 °C. The PCR amplification products were separated by stained (SafeView Biologicals, UK) 1.2% agarose gel electrophoresis at 100 V for 45 min and visualized on a UV transilluminator (BIO-RAD, USA). All the analysed samples gave amplicons of expected size, which were directly sequenced (BioFab Research, Roma, Italy). The resulting chromatograms were analysed and edited using the software Chromas version 2.33 (Technelysium Pty Ltd., South Brisbane, Australia). The sequences obtained were compared to those at the same gene previously deposited in GenBank and available at the website (http://www.ncbi.nlm.nih.gov/genbank/, accessed on 20 December 2020) by using the BLAST application.

### 5.4. Serological and Microscopical Analysis

In four cases, the patient’s serum was also available. Therefore, IgG antibodies against Onchocercidae filariae antigens were analysed using a commercial ELISA test (*Acanthocheilonema vitae*, Bordier Affinity Products, Crissier, CH, USA) following manufacturer’s protocol.

Finally, the modified Knott test [30] was performed to detect circulating microfilariae in the peripheral blood of all patients.

## Figures and Tables

**Table 1:** Demographic and clinical data of the cases described in this study

Case	Patient Gender	Age (Y)	Province	Site of Infection	Isolate	Species Identification
1	M	20	Rome	cheek	live worms	*D. repens*
2	F	56	Rome	lung	nodule	*D. repens*
3	F	55	Rome	lung	nodule	*D. repens*
4	F	70	Rome	calf muscle	live worms	*D. repens*
5	F	46	Rome	ocular conjunctiva	live worms	*D. repens*
6	F	-	Pisa	ocular conjunctiva	live worms	*D. repens*
7	F	-	Pescara	coccyx	nodule	*D. repens*
8	F	55	Latina	breast	nodule	*D. repens*
